# *Neoandracantha peruensis* n. gen. n. sp. (Acanthocephala, Polymorphidae) described from cystacanths infecting the ghost crab *Ocypode gaudichaudii* on the Peruvian coast

**DOI:** 10.1051/parasite/2017038

**Published:** 2017-10-25

**Authors:** Omar M. Amin, Richard A. Heckmann

**Affiliations:** 1 Institute of Parasitic Diseases, 11445 E. Via Linda, no. 2-419, Arizona, 85259 Scottsdale U.S.A; 2 Department of Biology, Brigham Young University, Utah, 84602 Provo U.S.A

**Keywords:** *Neoandracantha peruensis* n. gen. n. sp, description, EDAX analysis, *Ocypode gaudichaudii*, Peru coast

## Abstract

The cystacanths of *Neoandracantha peruensis* n. gen. n. sp. are described from the ghost crab *Ocypode gaudichaudii* collected from the Pacific coast of Peru. While it is uncommon to describe acanthocephalan taxa from immature stages, the presence of clear-cut distinguishing features separating the present material from its nearest congeneric taxa, and the absence of adults, justifies the erection *N. peruensis*. The new genus is distinguished by having three separate fields of trunk spines. Specimens of *N. peruensis* have a slender trunk with two anterior swellings, 3 separate fields of spines on the foretrunk swelling, and no genital spines on the hindtrunk. The proboscis of the new species is heavily armored with 21–22 longitudinal rows of 22 hooks each. Hook no. 14 is more robust ventrally than dorsally. Cystacanths of *N*. *peruensis* also have a long tubular hindtrunk and the males have diagonal testes in the midtrunk swelling. Specimens of the closely related *Andracantha* Schmidt, 1975 have anteriorly enlarged pear-shaped *Corynosoma*-like trunks, only two fields of anterior trunk spines with occasional genital spines, and bilateral or tandem testes. Proboscides of species of *Andracantha* have considerably fewer hooks that gradually decrease in size posteriorly. The taxonomic component of this work is amplified by metal analysis of hooks and spines that shows a marked amount of magnesium (Mg) in hooks but not in spines. The highest level of sulfur (S) was found in the outer layer of hooks and anterior spines. The metal footprint of hooks and spines varies in different species of acanthocephalans and has an interspecific diagnostic value.

## Abstract (fr)

Les cystacanthes de *Neoandracantha peruensis* n. gen. n. sp. sont décrits à partir de spécimens du crabe *Ocypode guadichaudi* recueillis sur la côte Pacifique au Pérou. Bien qu'il soit rare de décrire les taxons d'Acanthocéphales à partir de stades immatures, la présence de caractéristiques distinctives séparant clairement ce matériel de ses taxons congénères les plus proches, en l'absence d'adultes, justifie l'érection de *N. peruensis*. Le nouveau genre se distingue par l'existence de trois champs distincts d'épines sur le tronc. Les spécimens de *N. peruensis* ont un tronc mince avec deux renflements antérieurs, 3 champs d'épines séparés sur le renflement à l'avant du tronc et pas d'épines génitales sur l'arrière du tronc. Le proboscis de la nouvelle espèce est lourdement armé avec 21 à 22 lignes longitudinales de 22 crochets chacun. Le crochet numéro 14 est plus robuste ventralement que dorsalement. Les cystacanthes de *N. peruensis* ont également un tronc long et tubulaire et les mâles ont des testicules diagonaux dans le renflement du milieu du tronc. Les spécimens du genre proche *Andracantha* Schmidt, 1975 ont un tronc élargi à l'avant en forme de poire comme *Corynosoma*, seulement deux champs d'épines sur le tronc antérieur avec des épines génitales occasionnelles et des testicules bilatéraux ou en tandem. Les proboscis des espèces d'*Andracantha* ont beaucoup moins de crochets, qui diminuent graduellement en taille à l'arrière. La composante taxonomique de ce travail est renforcée par l'analyse des métaux des crochets et des épines, qui montre une quantité marquée de magnésium (Mg) dans les crochets mais pas les épines. Le plus haut niveau de soufre (S) a été trouvé dans la couche externe des crochets et des épines antérieures. L'empreinte métallique des crochets et des épines varie selon les différentes espèces d'Acanthocéphales et a une valeur diagnostique interspécifique.

## Introduction

The family Polymorphidae Meyer, 1931 includes a wide array of genera that parasitize aquatic birds and mammals. All genera have one thing in common: trunk spines in varied patterns. The confusion surrounding the separation of the various species into recognizable genera based on the trunk spine arrangements was resolved on the basis of the key to the genera in the family developed by Schmidt [29]. Of the genera recognized [29], only *Corynosoma* Lühe, 1904 has members with genital spines in one or both sexes, or only occasionally. As Schmidt [29] correctly mentioned, “….separate fields of trunk spines comprise another convenient character to separate genera, as has been done with *Diplospinifer* Fukui 1929 and *Southwellina* Witenberg 1932.” Schmidt [30] established the genus *Andracantha* to contain polymorphid species with two fields of trunk spines and genital spines in one or both sexes. The genital spines were noted to occasionally shift anteriorly or be absent. Genital spines were noted by their absence in *Andracantha mergi* (Lunström, 1941) Schmidt, 1973 and in *Andracantha tandemtesticulata* Monteiro, Amato, Amato, 2006. Aznar et al. [5] suggested that the absence of genital spines should not be construed as the sole criterion to exclude specimens from *Corynosoma* or *Andracantha*. We agree. Similarly, we are proposing to establish a new polymorphid genus and species with three fields of trunk spines and uncertain genital spines since all our specimens are immature.

The only background history relevant to the discovery of *Neoandracantha peruensis* n. gen., n. sp. collected from the ghost crab *Ocypode gaudichaudii* Milne-Edwards and Lucas off the Pacific coast of Peru at Callao is its earlier misdiagnosis and reporting once from the same host species in the same location as *Andracantha* sp. by Vasquez et al. [34] in a symposium abstract. Specimens of the new species may be limited in distribution compared to cystacanths of related polymorphids such as *Profilicollis altmani* (Perry, 1942) Van Cleave, 1947 that infect the sand crab Emerita analoga (Stimpson) also off the Peruvian coast [33], and elsewhere along the eastern Pacific and western Atlantic coasts of North America [31,10]. Attempts to find adults of *N. peruensis* in local cormorants have not been successful so far. Most species of the related genus *Andracantha* Schmidt, 1975 have piscivorous birds of the genus *Phalacrocorax* Brisson as their definitive hosts [20]. Presently, we aim to describe only the material at hand and will then proceed with further evaluations as new information becomes available. The independent metal analysis of proboscis hooks and trunk spines cut with a gallium beam (LIMS) made use of a dual-beam scanning electron microscope equipped with X-ray scanning (EDAX) to understand the biology of the attachment structures.

## Materials and methods

### Collections

We examined a total of 1,069 ghost crabs, weighing on average 14 g (males) and 17 g (females), and collected in 30 × 30 m grids from various beaches near Lima, Peru. Bióloga Asucena Naupay, Universidad Nacional Mayor de San Marcos, Dr. José Iannacone and his students, Universidad Nacional Federico Villareal, Lima, Peru, and their student assistants and collaborators [4,9,16,19,25,27] collected a total of 12 cystacanths of the new species (Table 1). Study areas were set in peripheral and coastal boundaries. Burrows were located, counted and measured. Crabs were collected and stored in Ziploc bags in 20 mL of 40% formalin for preservation until the hepatopancreas, intestinal surface, and body cavity were examined in the laboratory.

**Table 1:** Collections of specimens of *Neoandracantha peruensis* from the ghost crab *Ocypode gaudichaudii* from the Peruvian coast near Lima between 2011 and 2017.

Ghost crabs			Location	Geographical coordinates	Collectors	Reference No.	Date
					
Exam.	Infected	Worms					
60	4 (7.0%)	4	Ventanilla Callao	11° 51' 20” S, 77° 04' 25” W	Asucena Maupay	–	Nov. 2011
200	1 (0.5%)	4	Callao (3 beaches)	12° 2' S, 77° 8' W	Others*	–	Oct. 2015–Jan. 2016
90	0	0	San Pedro, Lurin	10° 25' 51” S, 76° 31' 03” W	Others*	–	July 2015
30	0	0	Chancay, Huaral	11° 85' 77” S, 77° 96' 57” W	Others*	–	July 2015
90	0	0	Santa Maria del Mar	12° 25' S, 76° 47' W	Others*	–	July 2015
30	0	0	Huara, Chancay	11° 55' 77” S, 77° 96' 57” W	Others*	–	July 2015
30	0	0	Venecia	12° 02' 46” S, 77° 02' 34” W	Others*	–	July 2015
50	0	0	Playa Manache, Huarmey	10° 04' 07” S, 78° 09' 47” W	Others*	–	Dec. 2015–Jan. 2016
50	0	0	Playa Colorado, Barranca	10° 46' 19” S, 77° 45' 35” W	Others*	–	Dec. 2015–Jan. 2016
50	0	0	Playa Chacra y Mar, Chancay	11° 37' 19” S, 77° 13' 51” W	Others*	–	Dec. 2015–Jan. 2016
50	0	0	Playa Gallard, Cerro Azul	13° 01' 57” S, 76° 29' 12” W	Others*	–	Dec. 2015–Jan. 2016
50	0	0	Playa la Isla, Barranca	10° 37' 69” S, 77° 41' 54” W	Others*	–	Dec. 2015–Jan. 2016
50	0	0	Playa Colorado	10° 46' 19” S, 77° 45' 35” W	Rosas et al.	27	Feb. 2017
50	1 (2.0%)	1	Playa Manache	10° 04' 07” S, 78° 09' 47” W	Quijón et al.	25	Feb. 2017
45	1 (2.2%)	1	Playa la Isla, Barranca	10° 37' 69” S, 77° 41' 54” W	Donayre et al.	9	Feb. 2017
50	2 (4.0%)	2	Playa Chacra y Mar	11° 37' 19” S, 77° 13' 51” W	Laura et al.	16	Feb. 2017
					Luis et al.	19	
94	0	0	Playa Gallardo	10° 46' 19” S, 77° 45' 35” W	Arcos et al.	4	Feb. 2017
1069	9 (0.8%)	12					

* Specimens were collected by student assistants and associates of Bióloga Asucena Naupay, Universidad Nacional Mayor de San Marcos and of Dr. José Iannacone, Universidad Nacional Federico Villareal, Lima, Peru.

Initial collections were made on three beaches of Callao district, located west of the Lima Metropolitan area and bordering Lima Province to the north, east, and south, and the Pacific Ocean to the west. Callao is the same locality from which Vasquez et al. [34] obtained a large number of cystacanths (189) from 178 ghost crabs between January and April 2012; they did not wish to make any of their specimens available for our examination.

Descriptions of some of the other collecting sites (Table 1) follow. Playa Colorado (Colorado Beach and intertidal sand); Playa Manache (poor presence of potential bird host populations); Playa la Isla (off the South Beach Island Barranca); Playa Chacra y Mar (a polluted site where *Ocypode* spp. are prevalent); and Playa Gallardo crabs were mostly (81%) juveniles. Overall, we had minimal success and the stochastic environment, climate and other ecological variables were implicated.

### Study of acanthocephalans

Worms were punctured with a fine needle and subsequently stained in Mayer's acid carmine, destained in 4% hydrochloric acid in 70% ethanol, dehydrated in ascending concentrations of ethanol (24 hr each), and cleared in 100% xylene then in 50% Canada balsam and 50% xylene (24 hr each). Whole worms were then mounted in Canada balsam. Measurements are in micrometers, unless otherwise noted; the range is followed by the mean values between parentheses. Width measurements represent maximum width. Trunk length does not include proboscis, neck, or bursa. Line drawing were created by using a Ken-A-Vision micro-projector (Ward's Biological Supply Co., Rochester, NY, USA), which uses cool quartz iodine 150 W illumination. Color-coded objectives, 10 X, 20 X, and 43 X lenses were used. Images of stained whole mounted specimens were projected vertically on 300 series Bristol draft paper (Strathmore, Westfield, MA, USA), then traced and inked with India ink. Projected images were identical to the actual specimens being projected. The completed line drawings were subsequently scanned at 600 pixels on a USB key and subsequently downloaded to a computer.

Type specimens were deposited at the University of Nebraska's State Museum's Harold W. Manter Laboratory (HWML) collection in Lincoln, Nebraska, USA.

### SEM (scanning electron microscopy)

Samples of parasites that had been fixed and stored in 70% ethanol were processed following standard methods [18]. These included critical point drying (CPD) in sample baskets and mounting on SEM sample mounts (stubs) using conductive double-sided carbon tape. Samples were coated with gold and palladium for 3 minutes using a Polaron #3500 sputter coater (Quorum (Q150 TES) www.quorumtech.com), establishing an approximate thickness of 20 nm. Samples were placed and observed in an FEI Helios Dual-Beam Nanolab 600 Scanning Electron Microscope (FEI, Hillsboro, OR, USA) with digital images obtained in the Nanolab software system (FEI, Hillsboro, OR, USA), and then transferred to a USB key for future reference. Images were taken at various magnifications. Samples were received under low vacuum conditions using 10 KV, spot size 2, 0.7 Torr using a GSE detector.

### X-ray microanalysis (XEDs), energy dispersive analysis for X-ray (EDAX)

Standard methods were used for preparation, similar to the SEM procedure. Specimens were examined and positioned with the above SEM instrument, which was equipped with a Phoenix energy-dispersive X-ray analyzer (FEI, Hillsboro, OR, USA). X-ray spot analysis and live scan analysis were performed at 16 KV with a spot size of 5 and results were recorded on charts and stored with digital imaging software attached to a computer. The TEAM *(texture and elemental analytical microscopy) software system (FEI, Hillsboro, OR, USA) was used. Data were stored on a USB key for future analysis. The data included weight percent and atom percent of the detected elements following correction factors.

### Ion sectioning of hooks

Typical hooks from the proboscis were cut in cross-sections at three levels: tip, middle, and base with a gallium beam (liquid ion metal source − LIMS). Other hooks were cut along the mid-longitudinal plane with the gallium beam and each cut was scanned twice with X-ray at two positions for the hook (edge and middle). The trunk has three spiny fields: anterior, middle, and posterior. Spines from each area were cut with the gallium beam along the cross-section and then scanned with X-ray for chemical elements. Data were stored on a USB key for future use.

A dual-beam SEM with a gallium (Ga) ion source (GIS) was used for the LIMS part of the process. The proboscis hooks were sectioned using a probe current between 0.2 nA and 2.1 nA according to the rate at which the area was cut. The time of cutting was based on the nature and sensitivity of the tissue. Following the initial cut, the sample also went through a milling process to obtain a smooth surface. The cut was then analyzed for chemical ions with an electron beam (Tungsten) to obtain an X-ray spectrum. Results were stored with the attached imaging software then transferred to a USB key for future use. The intensity of the GIS was variable due to the nature of the material being cut.

## Results and discussion

The following description is based on the study of 12 cystacanths obtained from 6 out of 1,069 examined ghost crabs (0.56%) from the Pacific coast near Lima, Peru with a maximum of 4 worms per crab. Ghost crabs, genus *Ocypode* Weber, inhabit the sandy shores of tropical and subtropical regions throughout the world. They are mostly nocturnal, inhabiting the deep burrows in the intertidal zone and are generalist scanvengers and predators of small animals. The ghost crab *O. gaudichaudii* is found along the Pacific coast of the Americas from Guatemala to Chile [14,28].

### *Neoandracantha* n. gen.

urn:lsid:zoobank.org:act:ACBEF202-8280-4E89-A03F-7B76C0BEAFA8

Diagnosis. Polymorphidae. Trunk in 3 segments, foretrunk, midtrunk, and hindtrunk; the first two separated by constriction. Foretrunk slender with 3 fields of spines and prominent middle swelling and including proboscis receptacle and lemnisci. Midtrunk bulbous with no spines and including testes in males and embryonic eggs at its posterior end in females. Hind trunk tubular including distal underdeveloped reproductive system, genital ligaments, and genital terminalia of both sexes. Proboscis with many longitudinal rows of many rooted hooks and unrooted spines; swollen near middle at level of transition between hooks and spines. Proboscis receptacle double-walled inserted at base of proboscis with cephalic ganglion near its middle. Testes diagonal. Cement glands barely discernable. Gonopores terminal in males and subterminal in females. Parasites of crabs off Peruvian Pacific.

Type species: *Neoandracantha peruensis* n. sp.

#### Remarks

Cystacanths of the genus *Neoandracantha* n. gen. characteristically have 3 fields of spines separated by bare zones in the slender foretrunk which has a middle swelling bearing the middle field of spines. The 3 fields of spines alone set the new genus apart from *Andracantha* which has only 2 fields of spines on the anterior trunk. The foretrunk and the bulbous spineless midtrunk of the new genus are separated by a constriction and the testes are diagonal compared to being in tandem or bilateral as they are in all 7 species of *Andracantha*.

### *Neoandracantha peruensis* n. sp.

urn:lsid:zoobank.org:act:A8A22A5A-05CC-40E3-BBDC-63164301F19B

Family: Polymorphidae Meyer, 1931

Genus: *Neoandracantha* n. gen.

Type host of cystacanths: *Ocypode gaudichaudii* Milne-Edwards and Lucas

Type locality: The Pacific Ocean off the Peruvian coast at Lima (12°2^′^36^″^S 77°1^′^42^″^W).

Site of infection: hepatopancreas and intestinal body cavity surface.

Type specimens: University of Nebraska's State Museum's Harold W. Manter Laboratory (HWML) collection in Lincoln, Nebraska, Collection No. 139135 (holotype male) and No. 139136 (allotype female).

Etymology: The name of the new genus addresses the relation to the genus *Andracantha*. The specific name describes the geographical location.

#### Description of cystacanths (Figures 1–23)

**Figures 1-6:**
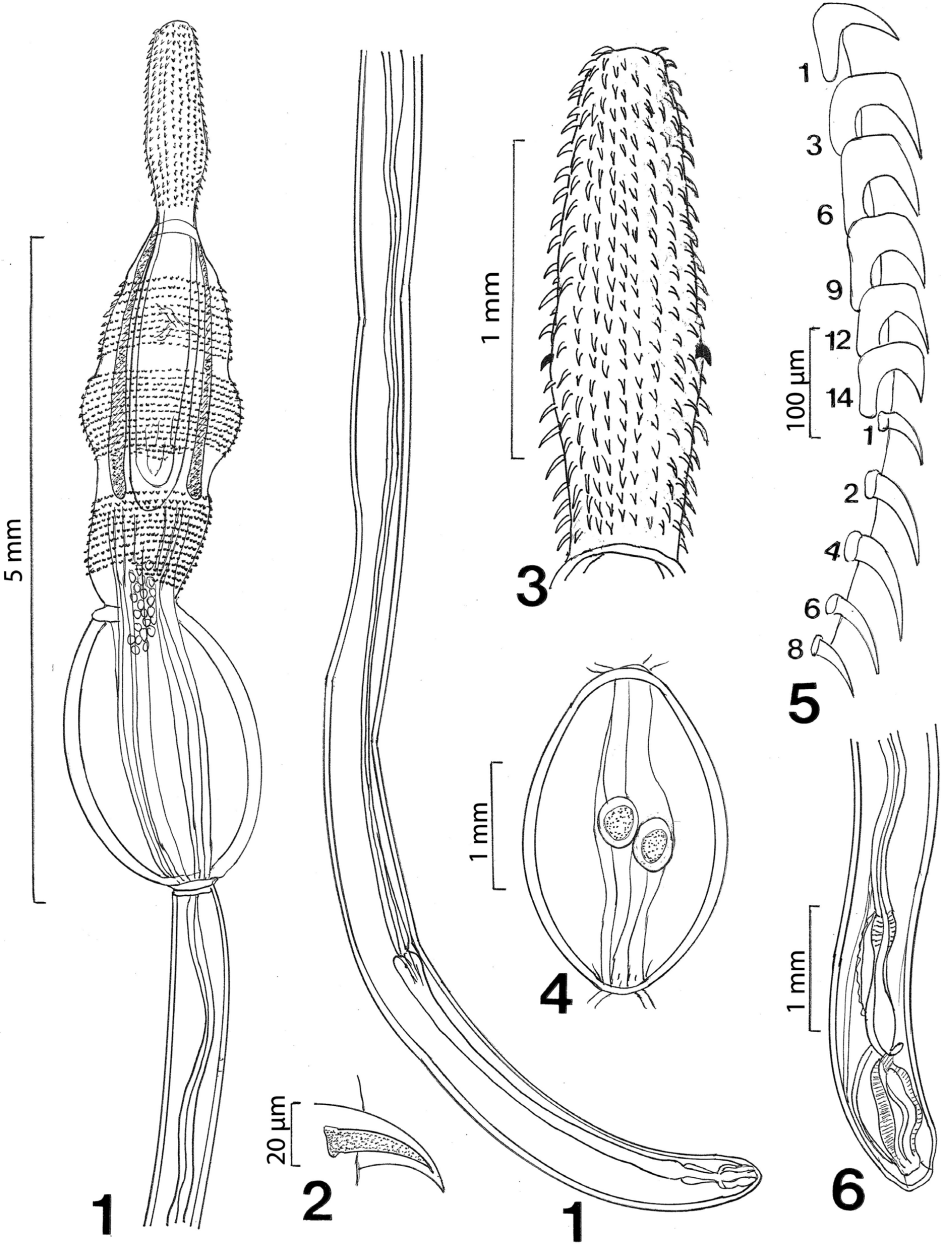
Line drawings of male and female cystacanths of *Neoandracantha peruensis* from ghost crabs, *Ocypode gaudichaudii*, from the Pacific Ocean off Peru. 1. Allotype female; note embryonic eggs between foretrunk and midtrunk and developing cephalic ganglion, female reproductive structures, and genital ligaments. 2. A trunk spine from the posterior field of foretrunk spines. 3. The proboscis of paratype female in Figure 1. Note the ventral robust hook no. 14 opposite the normal dorsal hook on the other side; boths hooks are blackened. This is a consistent characteristic of male and female specimens of *N. peruensis*. 4. The midtrunk of the holotype male showing the characteristic disposition of the diagonal testes. 5. One longitudinal row of selected representative hooks and spines numbered from anterior. 6. The posterior end of the hindtrunk of the male holotype showing the developing bursa and Saefftigen's pouch attached to the posterior end of the genital ligament which runs through the trunk.

**Figures 7-11:**
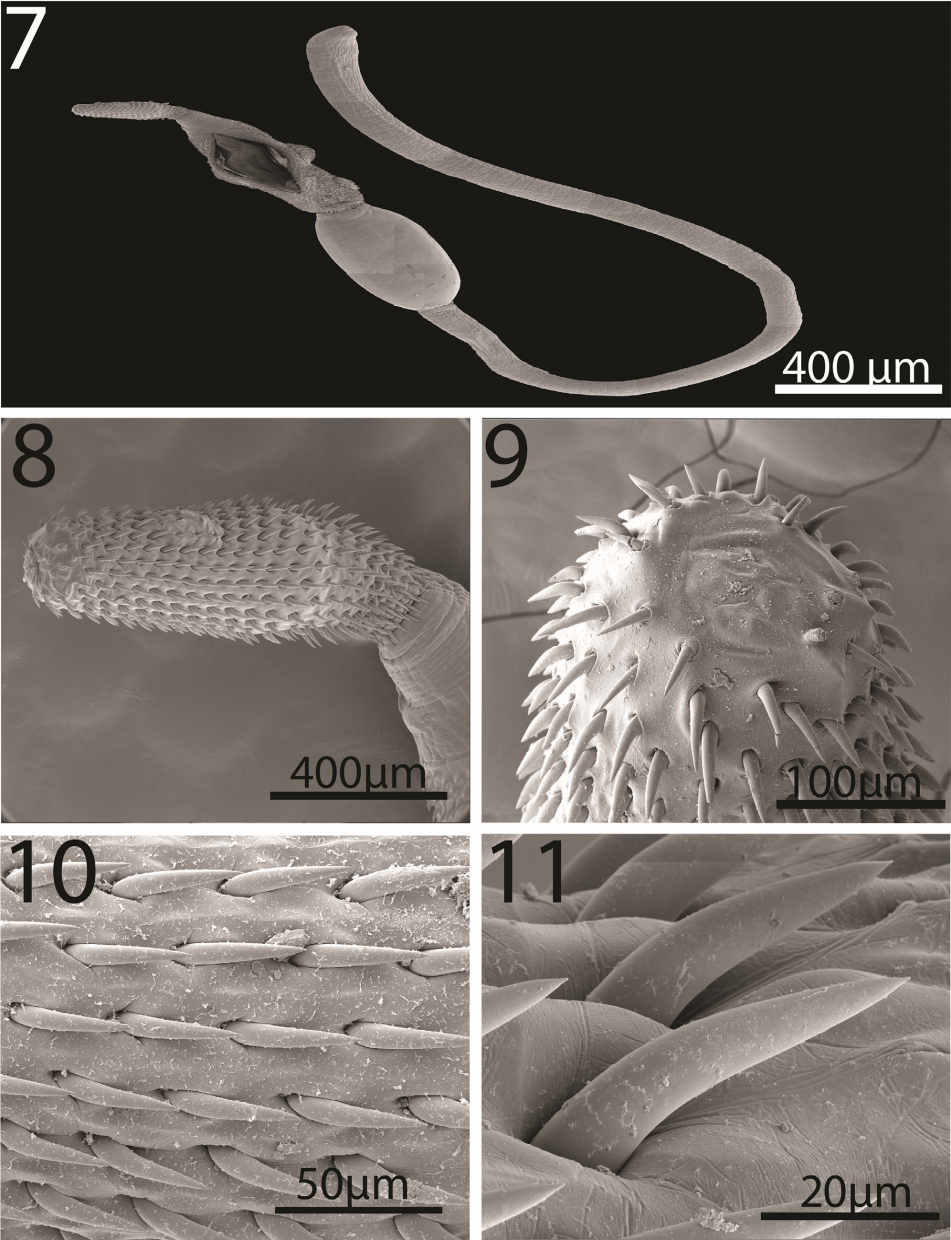
SEM of cystacanths of *Neoandracantha peruensis* from ghost crabs, *Ocypode gaudichaudii*, from the Pacific Ocean off Peru. 7. Allotype female. The foretrunk was slit open intentionally. 8. The proboscis of the allotype female in Figure 1. 9. The apical end of the proboscis showing its unarmed tip. 10. Proboscis hooks at the middle of the proboscis. 11. A few enlarged hooks showing their shape and orientation on the proboscis.

**Figures 12-17:**
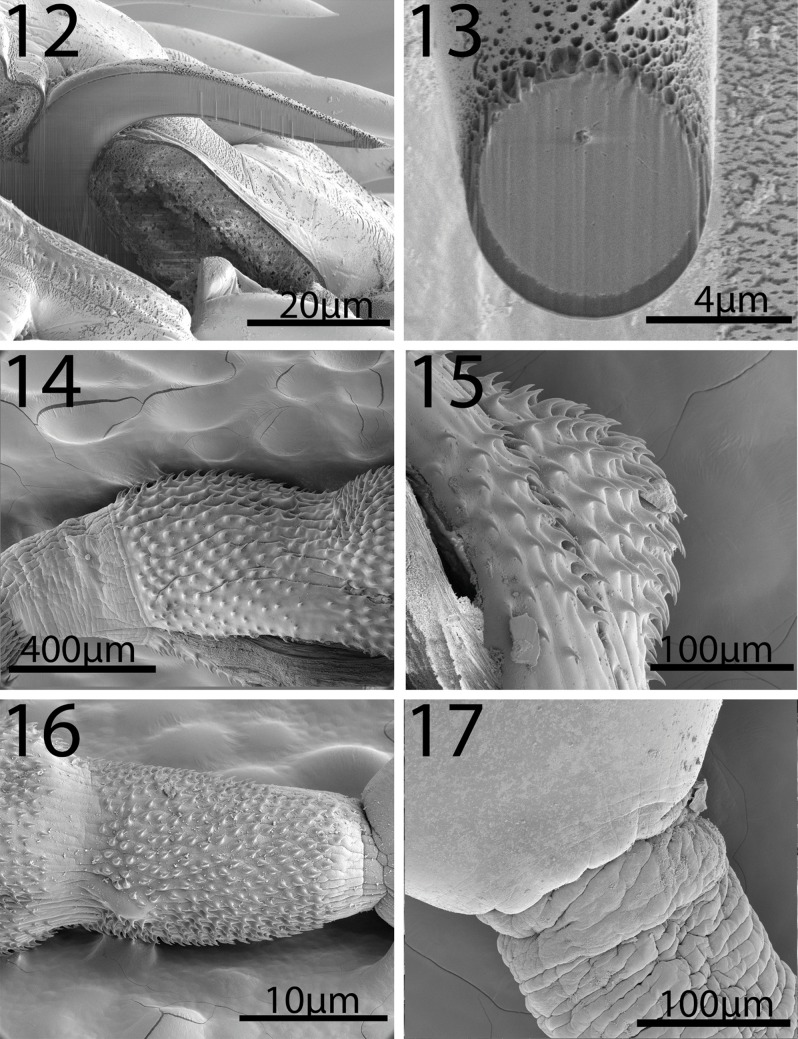
SEM of cystacanths of *Neoandracantha peruensis* from ghost crabs, *Ocypode gaudichaudii*, from the Pacific Ocean off Peru. 12–13. Lateral and cross-sections of gallium cut probosacis hooks showing their solid core and thin cortical layer. 14. The anterior field of spines of the foretrunk. 15. The middle field of spines of enlarged middle area of the foretrunk. 16. The posterior field of spines of the foretrunk near the junction with the midtrunk. 17. The unarmed junction of the midtrunk and hindtrunk.

**Figures 18-23:**
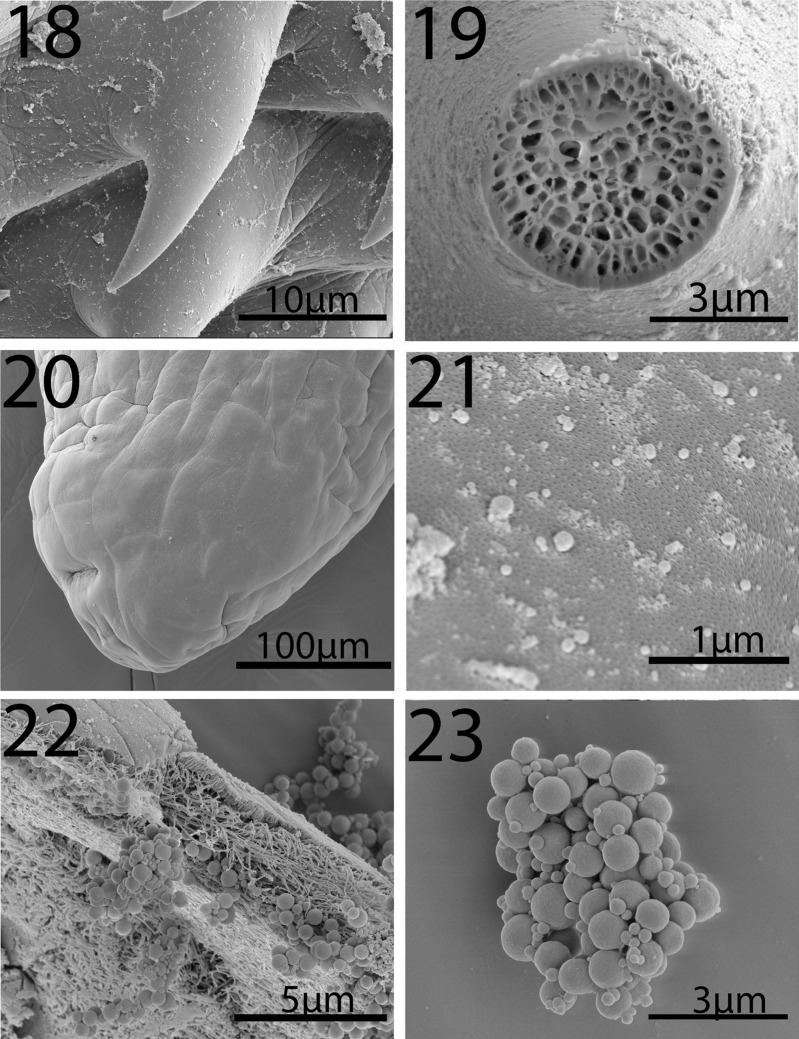
SEM of cystacanths of *Neoandracantha peruensis* from ghost crabs, *Ocypode gaudichaudii*, from the Pacific Ocean off Peru. 18. A spine from the posterior field of the foretrunk spines. 19. A gallium cut cross-section of a spine showing its spongy structure. 20. The posterior end of a female hindtrunk showing the subterminal position of the gonopore (lower left). 21. Micropores at the foretrunk of a female specimen. 22. Eggs in the body cavity of a female at the foretrunk-midtrunk junction. 23. A small cluster of eggs showing their different developmental stages.

General: With characters of the genus *Neoandracantha* (Polymorphidae). Structures usually relatively larger in females than in males. Trunk in 3 segments; anterior 2 segments (foretrunk and midtrunk) separated by constriction. Foretrunk with middle swelling enclosing proboscis receptacle and lemnisci and bearing 3 fields of spines separated by bare zones (Figures 1, 7, 14–17). Mid trunk ovoid, unarmed and includes testes in males (Figures 1, 4). Micropores in proboscis and two anterior trunk regions only. Hindtrunk (tail) tubular, slightly annulated, containing genital ligaments extending anteriorly into foretrunk and ending posteriorly into developing male and female reproductive terminalia (Figures 1, 4, 6). Hind trunk without micropores, occasionally withdrawn within midtrunk. Gonopore terminal in males (Figure 6) and subterminal in females (Figure 20). Trunk spines apparently less numerous in males than in females and most numerous in swollen middle field of foretrunk (Figure 15). Fields of spines may occasionally be incomplete and bare zones of separation may rarely be marginally traversed by occasional spines (Figures 14, 16, Table 3). Proboscis unarmed apically (Figure 9), with 20–22 hook and spine rows, cylindrical, widens at posterior third where anterior 14 robust rooted hooks transition into posterior 8 (rarely 9) slender rootless spines (Figures 3, 8). Hooks somewhat straight and sharply pointed posteriorly with solid massive core and thin cortical layer (Figures 5, 10–13). Spines arched and pointed posteriorly (Figure 18) with spongy core corresponding to cuticular micropores (Figure 19). Hook roots powerful and straight, not curved, slightly longer than hooks. Longest spines longer than longest hooks. Anterior-most spines and hooks shortest. Ventral posteriormost short hook no. 14 invariably considerably more robust than dorsal hook no. 14 on opposite side in males and females (Figure 3, Table 2). Proboscis occasionally not yet developmentally extruded from foretrunk. Proboscis receptacle double walled extending posteriorly just past foretrunk swelling between second and third fields of spines, with cephalic ganglion nerve elements near its middle. Lemnisci digitiform about as long as receptacle (Figure 1). Midtrunk bulbous including micropores (Figure 21) and 2 diagonal ovoid testes in males (Figure 4). Hindtrunk (tail) tubular containing genital ligaments extending anteriorly into foretrunk and ending posteriorly into developing male and female reproductive terminalia (Figures 1, 6). Tubular hind trunk without micropores, occasionally withdrawn within midtrunk. Gonopore terminal in males (Figure 6) and subterminal in females (Figure 20).

**Table 3:** Distribution and size of trunk spines of the foretrunk of 3 male and 3 female cystacanths of *Neoandracantha peruensis*.

	Anterior field of spines		Middle field of spines		Posterior field of spines	
			
	Males	Females	Males	Females	Males	Females
Dorsal spines						
Number	12–16 (14)^a^	12–15 (14)	8–10 (9)	11–12 (11)	3–17 (12)	4–13 (10)
Length	32–34 (33)^a^	36–37 (36)	32–52 (42)	45–55 (49)	37–62 (49)	47–75 (57)
Ventral spines						
Number	10–14 (12)	12–14 (13)	11–18 (15)	11–13 (12)	11–21 (16)	9–25 (16)
Length	32–34 (33)	42–45 (44)	37–52 (45)	50–55 (53)	37–52 (47)	55–60 (57)
Spines/circle	48–50 (49)	38–42 (40)	64–68 (66)	56–64 (60)	40–54 (46)	38–46 (43)

a Range (mean)

**Table 2:** Measurements of dorsal and ventral proboscis hooks and spines of 3 male and 3 female cystacanths of *Neoandracantha peruensis*.

	Males				Females			
		
	Dorsal		Ventral		Dorsal		Ventral	
				
	Length	Diameter	Length	Diameter	Length	Diameter	Length	Diameter
Hooks								
1	45–50 (47)^a^	10.12 (11)^a^	40–45 (42)	11–12 (11)	35–45 (40)	10–12 (11)	45–55 (50)	10–12 (11)
2	54–60 (57)	12–16 (14)	45–51 (48)	12–14 (13)	45–55 (50)	11–12 (11)	60–68 (64)	14–18 (16)
3	64–72 (68)	20–25 (22)	52–60 (56)	15–18 (16)	55–60 (57)	12–18 (15)	74–75 (74)	17–20 (18)
4	73–75 (74)	20–23 (21)	60–70 (65)	18–20 (19)	62–70 (66)	17–20 (18)	72–75 (73)	17–22 (19)
5	70–72 (71)	20–25 (22)	67–72 (69)	21–22 (21)	65–72 (68)	20–21 (20)	74–75 (74)	20–22 (21)
6	67–75 (71)	20–27 (23)	70–75 (72)	21–22 (21)	67–68 (67)	20–23 (21)	75–80 (77)	22–23 (22)
7	67–72 (69)	20–25 (22)	67–77 (72)	22–25 (23)	72–75 (73)	22–23 (22)	70–80 (75)	24–25 (24)
8	75–77 (76)	22–27 (24)	75–80 (77)	25–27 (26)	72–75 (73)	24–25 (24)	65–82 (73)	24–25 (24)
9	75–77 (76)	22–32 (27)	72–87 (79)	25–27 (26)	72–75 (73)	25–27 (26)	70–82 (76)	24–25 (24)
10	75–82 (78)	25–30 (27)	77–87 (82)	25–27 (26)	77–80 (78)	25–27 (26)	65–82 (73)	25–26 (25)
11	72–82 (77)	25–32 (28)	75–87 (81)	26–27 (26)	80–82 (81)	27–28 (27)	70–82 (76)	25–27 (26)
12	62–87 (74)	30–32 (31)	75–92 (83)	27–30 (28)	82–85 (83)	29–30 (28)	77–82 (79)	30–32 (31)
13	62–77 (69)	27–30 (28)	77–100 (88)	32–35 (33)	77–78 (77)	27–30 (28)	87–88 (87)	31–32 (31)
14	62–65 (63)	21–22 (21)	72–90 (81)	30–31 (30)	57–72 (64)	25–27 (26)	77–78 (77)	30–32 (31)
Spines								
1	57–60 (59)	10–15 (12)	50–60 (55)	15–20 (17)	47–67 (57)	12–17 (14)	45–62 (53)	15–17 (16)
2	85–87 (86)	15–17 (16)	82–85 (83)	14–15 (14)	85–92 (88)	15–17 (16)	92–95 (93)	18–20 (16)
3	95–96 (95)	15–20 (17)	90–100 (95)	15–17 (16)	87–90 (88)	16–17 (16)	82–102 (92)	17–18 (17)
4	95–96 (95)	15–20 (17)	90–100 (95)	15–17 (16)	90–91 (90)	17–18 (17)	80–97 (88)	15–17 (16)
5	90–95 (92)	17–18 (17)	82–87 (86)	14–15 (14)	87–88 (87)	18–19 (18)	80–102 (91)	15–20 (17)
6	77–92 (84)	15–16 (15)	75–82 (78)	15–16 (15)	77–87 (82)	15–17 (16)	70–72 (71)	14–15 (14)
7	75–85 (80)	15–17 (16)	72–75 (73)	14–15 (14)	67–77 (72)	14–15 (14)	55–67 (61)	14–15 (14)
8	62–70 (66)	14–15 (14)	57–62 (59)	14–15 (14)	57–62 (59)	12–15 (13)	45–55 (50)	10–15 (12)

a Range (mean) length and diameter at base in μm.

*Male* (based on 5 whole mounts and 1 specimen used for SEM generation): Trunk in 3 regions measuring 13.95–17.30 (15.49) mm in total length. Foretrunk 2. 02–2.92 (2.36) mm long by 0.63–1.15 (0.86) mm wide at middle swelling. Midtrunk 2.12–2.77 (2.38) mm long by 1.00–1.60 (1.21) mm at swelling. Tubular hind trunk 9.50–12.00 (10.75) mm long by 0.35–0.50 (0.42) mm wide at posterior end. See Table 2 for measurements and numbers of spines in 3 foretrunk fields. Proboscis 1.40–1.66 (1.58) mm long by 0.40–0.45 (0.43) mm wide at swelling. Most ventral hooks, especially posterior hooks and hook no. 14 from anterior, larger than dorsal hooks. Anterior-most and posterior-most spines shortest. See Table 2 for measurements of length and diameter at base of dorsal and ventral hooks and spines. Neck 416 long by 416 wide. Proboscis receptacle 1.22–2.31 (1.92) mm long by 0.40–0.75 (0.50) mm wide. Lemnisci equal, digitiform 0.99–2.18 (1.52) mm long by 0.07–0.16 (0.11) mm wide. Testes in midtrunk about equal (Figure 4). Anterior testis 364–426 (392) long by 260–406 (330) wide. Posterior testis 364–426 (395) long by 260–374 (314) wide. Developing retracted bursa and Saefftigen's pouch contained in male terminalia near posterior end of hindtrunk (tail): 10.50 mm long by 0.32–0.55 (0.43) mm wide (Figure 6).

*Female* (based on 5 whole mounts and 1 specimen used for SEM generation): Trunk in 3 regions measuring 14.50–18.60 (16.27) mm in total length. Foretrunk 2.72–2.87 (2.79) mm long by 0.93–1.17 (1.01) mm wide at middle swelling. Midtrunk 2.12–2.30 (2.23) mm long by 1.00–1.50 (1.19) mm wide at swelling. Tubular hind trunk 10.16–13.00 (11.25) mm long by 0.41–0.62 (0.50) mm wide at posterior end. See Table 2 for measurements and numbers of spines in 3 foretrunk fields. Proboscis 1.35–1.55 (1.47) mm long by 0.40–0.47 (0.43) mm wide at swelling. Most ventral hooks, especially posterior hooks and hook no. 14 from anterior, larger than dorsal hooks. Anterior-most and posterior-most spines shortest. See Table 2 for measurements of length and diameter at base of dorsal and ventral hooks and spines. Proboscis receptacle 2.20–2.45 (2.32) mm long by 0.44–0.55 (0.46) mm wide. Lemnisci equal, digitiform 1.87–2.29 (2.08) mm long by 0.10–0.15 (0.12) mm wide. Developing vagina, uterus, and uterine bell discernible at posterior end of genital ligament in hindtrunk (tail) (Figure 1). Embryonic eggs at various stages of development (Figures 22, 23) at junction between foretrunk and midtrunk (Figures 1, 22). Hind trunk: 10.80–10.87 (10.83) mm long by 0.45–0.47 (0.46) mm wide.

#### Remarks

*Neoandracantha peruensis* n. gen., n. sp. is primarily distinguished from species of the closely related *Andracantha* Schmidt, 1975 by having a slender trunk with two anterior swellings separated with a constriction, 3 prominent fields of spines on the foretrunk swelling separated by aspinose zones, and no genital spines. Adults and cystacanths of most species of *Andracantha* have anteriorly enlarged pear-shaped *Corynosoma*-like trunks, only two fields of anterior trunk spines, and occasional genital spines. In addition, cystacanths of *N. peruensis* have a long tubular posterior trunk and males have diagonally positioned testes in the midtrunk swelling compared to either bilateral or tandem testes in species of *Andracantha*. *Andracantha tandemtesticulata* described from the Neotropical cormorant, *Phalacrocorax brasilianus* (Gmelin) in Southern Brazil [20] is the only species of *Andracantha* that is close to *N. peruensis* in trunk shape and organization. Nevertheless, it has two fields of spines in the anterior trunk, tandem testes and different proboscis armature.

### Differences in parasite recovery

The discrepancy between our limited parasite recovery success compared to that of Vasquez et al. [34] who obtained 189 cystacanths from 24% of 178 ghost crabs examined between January and April 2012 is noteworthy. Such discrepancies are not uncommon. For instance, Schmidt and MacLean [31] reported 4 and 19 rock crabs *Cancer irroratus* Say infected with cystacanths of *Profilicollis major* Lundström, 1942 from 20 and 51 examined crabs; prevalence of 20% and 37%, respectively. Their subsequent examination of 700 and 350 rock crabs from the New Jersey and Delaware coasts over a period of 4 years yielded no parasites. For a better understanding of the distribution and habitats of populations of *O. gaudichaudii*, see Quijón et al. [25] and Moscoso [22].

### Cystacanth and adult comparisons

We attempted unsuccessfully to find adults of the new acanthocephalan species in various shore birds including the snowy egret, *Egretta thula* (Molina), Guanay cormorant, *Leucocarbo bougainvillii* (Lesson), royal tern, *Thalasseus maximus* (Boddaert), American oystercatcher, *Haematopus palliates* Temminck, and Franklin's gull *Leucophaeus pipixcan* (Wagler). We, however, believe that adults of *Neoandracantha peruensis* are similar to the described cystacanths based on corroborating reports. For example, Nickol et al. [23] described other polymorphid cystacanths of *Arhythmorhynchus frassoni* (Molin, 1858) Lühe, 1911 from fiddler crabs, *Uca rapax*, in Florida similar to our cystacanths of *N. peruensis*. Their specimens had an anterior spined foretrunk “ending in contriction followed by unspined bulbous swelling …. followed by long threadlike hindtrunk” (their Figure 1). They [23] further indicated that “The proboscis size, shape, and armature, including length of the hooks, of *A. frassoni* cystacanths are identical to those of adults.”

Comparable findings were found in male and female cystacanths of *Profilicollis botulus* (Van Cleave, 1916) Witenberg, 1932 with similar morphology to cystacanths of *N. peruensis* including “two trunk regions separated by a constriction with spiny anterior” from the hairy shore crab *Hemigrapsus oregonensis* (Dana) from British Columbia, Canada [8]. Ching [8] also reported that “the number of rows and hooks and shapes and proportions of the hooks are similar in cystacanths from shore crabs and (bottle-shaped) adults from the common gloden eye diving duck *Bucephalus clangula* (L.).” Other polymorphid cystacanths and adults with similar proboscis armature include *Corynosoma stanleyi* Smales, 1986 which was reported from 3 species of Australian shore crabs (*Paragrapsus gaimardii* Milne Edwards, *P. quadridentatus* Milne Edwards, *Cycloprapsus granulosus* Milne Edwards), and from one species of mammal, the water rat *Hydromys chrysogaster* Geoffroy, respectively [24]. Similarly, Brockerhoff and Smales [7] demonstrated matching proboscis armature and trunk spination, among other features, between cystacanths and adults of *Profilicollis novaezalnandensis* Brockerhoff and Smales, 2002 from the intertidal crab *Hemigrapsus crenulatus* (Milne Edwards) and adults from the oystercatcher *Haematopus ostralegus* *finschi* Martins in New Zealand.

In non-polymorphid acanthocephalans, “No significant differences were detected in proboscis length and hook length (Leidy, 1850) Schmidt, 1972 between cystacanths and adults of “*Macracanthorhynchus ingens* (Linstow, 1879) Meyer, 1932 and *Oligacanthorhynchus tortuosa* (Laidy, 1850) Schmidt, 1972. Hook size and proboscis length appear to remain stable through development from cystacanth to adult” [26]. Moore [21] asserted that the proboscis and hook morphometrics of *Mediorhynchus grandis* Van Cleave, 1916 are fixed by the time worms became infective cystacanths, and Amin [1] reported complete correspondence in all structures of developed cystacanths and adults of *Acanthocephalus dirus* Van Cleave (1931), Van Cleave and Townsend, 1936.

### Description of immature acanthocephalans

While it is uncommon to describe acanthocephalan taxa from immature stages, the presence of clear-cut distinguishing features, especially trunk spination and proboscis armature, separating the present material from its nearest congeneric taxa, in the absence of adults, justifies the erection of *N. peruensis* n. gen., n. sp. Other species of Acanthocephala have also been described from cystacanths collected from intermediate or paratenic hosts. For example, *Corynosoma beaglense* Laskowski, Jeżewski, Zdzitowiecki, 2008 was described from the cystacanth stage infecting the body cavity of *Champsocephalus esox* Günther (Perciformes) in the Beagle Channel [15]; the definitive hosts and adults remain unknown. Comparable cases can be drawn from the taxonomic literature on other helminth groups such as trypanorhynchid cestodes. For example, it is commonly accepted to describe new genera and species of trypanorhynchids from larvae because the taxonomy is based on the spines of the tentacles, which are the same in adults and larvae. For example, the original description of *Nybelinia surmenicola* Okada in Dollfus, 1929 (Tentacularidae: Trypanorhynchidae) *was made from plerocercoids* collected from the squid *Todarodes pacificus*, Steenstrup [17]. Thirty-six species of trypanorhynchid cestodes have been identified from plerocercoids, plerocerci, and merocercoids in actinopterygians, decapod crustaceans, bivalves, and gastropods in the Gulf of Mexico [13]. Many more such studies are reported from all over the world.

### X-ray microanalysis (XEDs), energy dispersive analysis for X-ray (EDAX)

**Figure 24:**
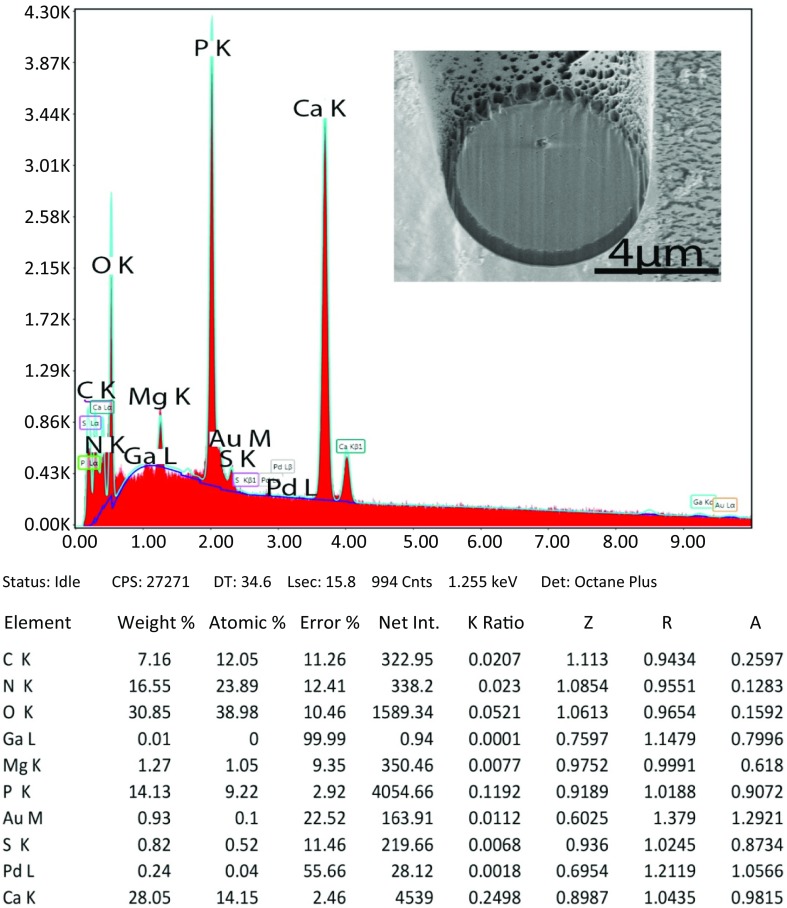
The printout for the elemental scan (EDXA) of a hook near the middle of the proboscis of *Neoandracantha peruensis*. Note values for all levels of the cut hooks in Table 4.

The metal profile of hooks and spines in species of Acanthocephala has a taxonomic implication as it will vary by species and can be used as an additional interspecific diagnostic tool. Results of the analysis of mineral content in proboscis hooks are found in Figure 24 and Table 4. The metal profile of hooks has been reported previously for *Echinorhynchus baeri* Kostylew, 1928 [2] and *Rhadinorhynchus oligospinosus* Amin and Heckmann, 2017 [3], among other species of acanthocephalans. In *N. peruensis*, the magnesium (Mg) appeared in marked amounts. Common elements (C and O) were recorded with Mg, P, S, and Ca appearing in the hooks. The highest level of sulfur (S) was found in the outer layer of hooks (edge layer) especially at the middle of the hook. These elements are critical for the mineralization of the hook which creates the hardened outer layer, an apatite, similar to tooth enamel for mammals. Mg probably plays a role in the mineralization of hooks similar to the disulfide bonds formed by S in the protein apatite.

**Table 4:** Chemical elements for 3 levels (tip, middle, base) of gallium cut hooks of *Neoandracantha peruensis*.^a^

	Hook edge		Hook center	
		
	Weight %	Atomic %	Weight %	Atomic %
Hook tip				
Magnesium (Mg)	1.02	0.84	1.08	0.60
Phosphorus (P)	12.70	8.25	15.28	10.70
Sulfur (S)	2.50	1.50	1.05	0.75
Calcium (Ca)	26.41	13.17	34.31	18.56
Hook middle				
Mg	0.26	0.21	0.93	0.81
P	7.84	4.77	14.91	10.28
S	6.20	3.64	0.28	0.19
Ca	20.80	9.78	34.03	18.14
Hook base				
Mg	0.75	0.69	0.82	0.74
P	14.79	10.65	14.70	10.53
S	0.32	0.22	0.20	0.14
Ca	36.92	20.55	37.23	20.61

a List includes four critical elements for the hardening of hooks (Mg, P, S, Ca). The other elements, carbon (C) and oxygen (O) are common in all living animals; gold (Au) and palladium (Pd) are used to coat the specimen in an 18 nm layer. Gallium (Ga) is the ion used to cut the specimen.

**Figure 25:**
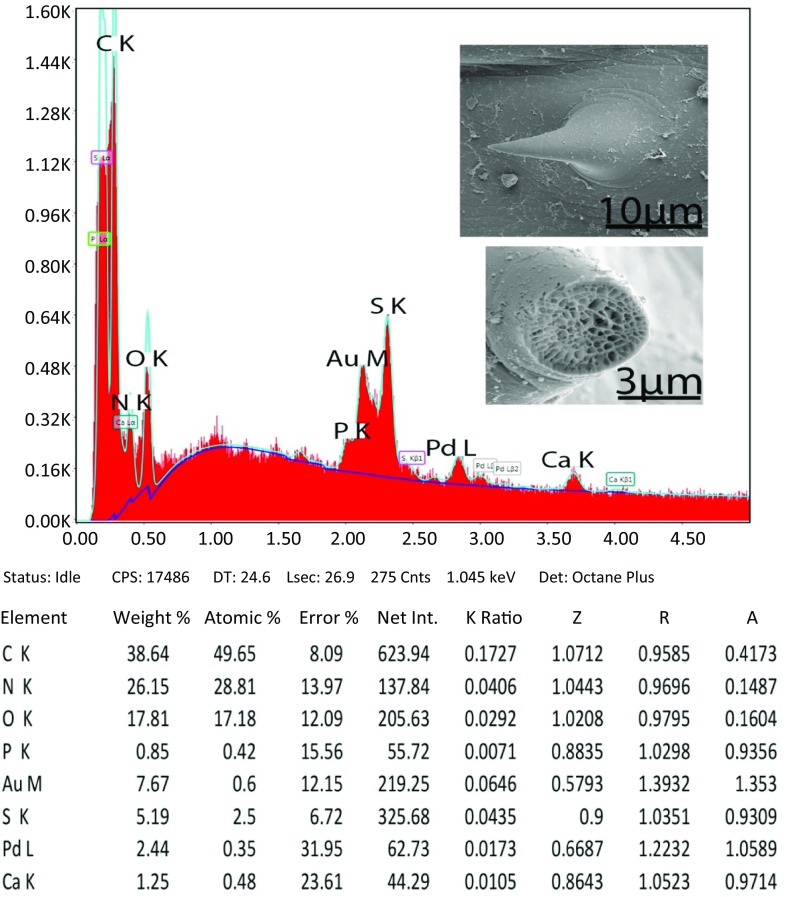
The printout for the elemental scan (EDXA) of a spine from the posterior field of spines in the foretrunk of *Neoandracantha peruensis*. Note values for spines in the other 2 fields in Table 5.

The data for the cut trunk spines using X-ray analysis (EDAX) can be found in Figure 25 and Table 5. Along with common chemical elements (C, O), the spines contained recordable amounts of phosphorus (P), calcium (Ca), and especially sulfur (S). There was an appreciable increase in S for the anterior spines, decreasing posteriorly. Thus, the more hardened spines are found in the anterior part of the trunk.

**Table 5:** Chemical elements (Ca, S, P) detected in cut spines from the anterior, middle and posterior fields of the foretrunk of *Neoandracantha peruensis*.^a^

	Weight %	Atomic %
Spines in anterior field		
Calcium (Ca)	1.25	0.48
Sulfur (S)	5.19	2.50
Phosphorus (P)	0.85	0.42
Spines in middle field		
Ca	0.7	0.27
S	3.55	1.72
P	0.53	0.27
Spines in posterior field		
Ca	0.49	0.22
S	2.82	1.59
P	0.57	0.30

a Other elements, carbon (C) and oxygen (O) are common in all living animals; gold (Au) and palladium (Pd) are used to coat the specimen in an 18 nm layer. Gallium (Ga) ions are used to cut the specimen.

## Conclusions

Polymorphid genera have trunk spines in varied patterns and genera are recognized based on the trunk spine arrangements [29]. Of the genera recognized by Schmidt [29], only *Corynosoma* Lühe, 1904 has one field of trunk spines and possibly genital spines in one or both sexes, or only occasionally. Schmidt [30] established the genus *Andracantha* to contain polymorphid species with two fields of trunk spines and genital spines in one or both sexes, if at all. Aznar et al. [5] suggested that the absence of genital spines should not be construed as the sole criterion to exclude specimens from *Corynosoma* or *Andracantha*. Our present contribution expands the concepts of polymorphids with one field of spines in *Corynosoma* to polymorphids with two fields of spines in *Andracantha* to polymorphids with three fields of spines in *Neoandracantha* with uncertain genital spines, since all our specimens are immatures. Structures other than trunk spines such as proboscis armature and placement of testes also contribute to the distinction of *Neoandracantha* n. genus from other polymorphids. We have also shown that size, number and distribution of trunk and proboscis armature in the cystacanths will match those in the adults should any be successfully recovered from their potential bird definitive hosts at a future date. We will continue our efforts to obtain adults from crab-eating birds in the same general areas where cystacanths were collected.

For the first time, we observed recordable amounts of magnesium in the proboscis hooks. Brazova et al. [6] had similar results for hooks of *Acanthocephalus lucii* (Müller, 1776). In our other studies [11,12,32], including phase map studies with X-ray of proboscis hooks, Mg was not detected in recordable amounts. The hardness of hook outer layers and anterior spines are demonstrated by their higher levels of sulfur.

### Conflict of interest

The authors declare that they have no conflict of interest.

## References

[1] Amin OM. 1982. Description of larval Acanthocephalus parksidei Amin, 1975 (Acanthocephala, Echinorhynchidae) from its isopod intermediate host, in *Proceedings of the Helminthological Society of Washington*, 49, 235-245.

[2] Amin OM, Heckmann RA, Evans RP, Tepe Y. 2016 A description of *Echinorhynchus baeri* Kostylew, 1928 (Acanthocephala: Echinorhynchidae) from *Salmo trutta* in Turkey, with notes on synonymy, geographical origins, geological history, molecular profile, and X-ray microanalysis. Parasite, 23, 56. 27991414 10.1051/parasite/2016067 PMC5178380

[3] Amin O.M., Heckmann RA. 2017 *Rhadinorhynchus oligospinosus* n. sp. (Acanthocephala, Rhadinorhynchidae) from mackerels in the Pacific Ocean off Peru and related rhadinorhynchids in the Pacific, with notes on metal analysis. Parasite, 24, 19. 28593837 10.1051/parasite/2017022 PMC5467225

[4] Arcos F, Chucos G, De la Torres J, Liza A, Medina H. 2017. Relaciones métricas y de población del Cangrejo Carretero *Ocypode gaudichaudii* (H. Milne Edwards & Lucas, 1843) en Playa Gallardo, Cerro Azul − Cañete. Report to Facultad de Ciencias Naturales y Matemática de la Universidad Nacional Federico Villarreal (UNFV) Jr. Río Chepén s/n El Agustino, Lima, Perú, p. 5.

[5] Aznar FJ, Beron-Vera B, Crespo EA, Raja JF. 2002 Presence of genital spines in a male *Corynosoma cetaceum* Johnston and Best, 1942 (Acanthocephala). Journal of Parasitology, 88, 403-404. 12054020 10.1645/0022-3395(2002)088[0403:POGSIA]2.0.CO;2

[6] Brazova T, Poddubnaya LG, Miss NR, Hanzelova V. 2014 Ultrastructure and chemical composition of the proboscis hooks of *Acanthocephalus lucii* (Müller, 1776) (Acanthocephala: Palaeacanthocephala) using X-ray elemental analysis. Folia Parasitologica, 6, 549-557. 25651697

[7] Brockerhoff AM, Smales LR. 2002 *Profilicollis novaezalnandensis* n. sp. (Polymorphidae) and two other acanthocephalan parasites from shore birds (Haematopodidae and Scolopacidae) in New Zealand, with records of two species in intertidal crabs (Decapoda: Grapsidae and Ocypodidae). Systematic Parasitology, 52, 55-65. 12023562 10.1023/a:1015011112900

[8] Ching HL. 1989 *Profilicollis botulus* (Van Cleave, 1916) from diving ducks and shore crabs of British Columbia. Journal of Parasitology, 75, 33-37. 2918442

[9] Donayre R, Olivera A, Ponce H, Quispe J. 2017 Análisis parasitológico del cangrejo carretero *Ocypode gaudichaudii* (Ocypodidae) en el tramo sur de la playa “la isla”, barranca, lima.Laboratorio de Ecología y Biodversidad Animal. Report to Facultad de Ciencias Naturales y Matemática. Lima: Universidad Nacional Federico Villarreal. Calle Río Chepén s/n, El Agustino, p. 16.

[10] Goulding TC, Cohen CS. 2014 Phylogeography of a marine acanthocephalan: lack of cryptic diversity in a cosmopolitan parasite of mole crabs. Journal of Biogeography, 1-12.

[11] Heckmann RA, Amin OM, Standing MD. 2007 Chemical analysis of metals in Acanthocephalans using energy dispersive x-ray analysis (EDXA, XEDS) in conjunction with a scanning electron microscope (SEM). Comparative Parasitology, 74, 388-391.

[12] Heckmann RA, Amin OM, Radwan NAE, Standing MD, Eggett DL, El Naggar AM. 2012 Fine structure and energy dispersive X-ray analysis (EDXA) of the proboscis hooks of *Rhadinorhynchus ornatus*, Van Cleave 1918 (Rhadinorhynchidae: Acanthocephala). Scientia Parasitologica, 13, 37-43.

[13] Jensen, K. 2009 Cestoda (Platyhelminthes) of the Gulf of Mexico, in Gulf of Mexico–Origins, Waters, and Biota. Biodiversity, Felder D.L., Camp D.K. Editors. College Station, Texas: Texas A&M University Press, p. 1393.

[14] Karleskint G, Turner RK, Small J. 2009. Intertidal communities. Introduction to Marine Biology (3^rd^ ed.). Cengage Learning, 356-411.

[15] Laskowski Z, Jeżewski W, Zdzitowiecki K. 2008 Cystacanths of Acanthocephala in notothenioid fish from the Beagle Channel (sub-Antarctica). Systematic Parasitology, 70, 107-117. 18427957 10.1007/s11230-008-9131-0

[16] Laura QC, Maslucán Guevara F, Melgarejo Espinoza Y, Kristhie PR, Alexandra QH. 2017 Biometría y fauna parasitológica de *Ocypode gaudichaudii* Milne-Edwards & Lucas, 1843 recolectados en la playa Manache Huarmey. Report to Facultad de Ciencias Naturales y Matemática. Lima: Universidad Nacional Federico Villarreal. Calle Río Chepén s/n, El Agustino, p. 10

[17] Lee JY, Kim JW, Park GM. 2016 Plerocercoids of *Nybelinia surmenicola* (Cestoda: Tentacularidae) in Squids, *Todarodes pacificus*, from East Sea, the Republic of Korea. Korean Journal of Parasitology, 54, 221-224. 27180583 10.3347/kjp.2016.54.2.221 PMC4870981

[18] Lee RE. 1992 Scanning electron microscopy and X-Ray microanalysis. Englewood Cliffs, New Jersey: Prentice Hall p. 458.

[19] Luis AY, Conny GC, Magdalena MC, Noemí MM. 2017 Estructura poblacional del *Ocypode gaudichaudii* y su relación con los Acantocephalos en la playa Chacra Y Mar (Chancay, Huaral-Perú). Report to Facultad de Ciencias Naturales y Matemática, Lima, Perú: Universidad Nacional Federico Villarreal, Jr. Rio Chepén S/n − El Agustino, p. 11.

[20] Monteiro CM, Amato JFR, Amato SB. 2006 A new species of *Andracantha* Schmidt (Acanthocephala: Polymorphidae) parasite of tropical cormorants, *Phalacrocorax brasilianus* (Gmelin) (Aves: Phalacrocoridae) from southern Brazil. Revista Brasileira de Zoologia, 23, 807-812.

[21] Moore DV. 1962 Morphology, life history, and development of the acanthocephalan *Mediorhynchus grandis* Van Cleave, 1916. Journal of Parasitology, 48, 76-86.

[22] Moscoso V. 2012. Catálogo de crustáceos decápodos y estomatópodos del Perú. Boletín Instituto del Mar del Perú (IMARPE), 27 (1-2), 1–209.

[23] Nickol BB, Heard RW, Smith NF. 2002 Acanthocephalans from crabs in the southeastern U.S., with the first intermediate hosts known for *Arhythmorhynchus frassoni* and *Hexaglandula Corynosoma*. Journal of Parasitology, 88, 79-83. 12053984 10.1645/0022-3395(2002)088[0079:AFCITS]2.0.CO;2

[24] Pichelin S, Kuris AM, Gurney R. 1998 Morphological and biological notes on *Polymorphus* (*Profilicollis*) *sphaerocephalus* and *Corynosoma Stanleyi* (Polymorphidae: Acanthocephala). Journal of Parasitology, 84, 798-801. 9714213

[25] Quijón P, Jaramillo E, Contreras H. 2001 Distribution and habitat structure of *Ocypode gaudichaudii* H. Milne Edwards & Lucas, 1843, in sandy beaches of Northern Chile. Crustaceana, 74, 91-103.

[26] Richardson DJ. 2005 Identification of cystacanths and adults of *Oligacanthorhynchus tortuosa*, *Macracanthorhynchus ingens*, and *Macacanthorhynchus hirudinaceus* based on proboscis and hook morphometrics. Journal of the Arkansas Academy of Science, 59, 205-209.

[27] Rosas B., Sarmiento A, Torres F, Vega K, Villasante N. 2017 Análisis biométrico y dispersión parasitaria en *Ocypode gaudichaudii* de la playa Colorado, distrito de Barranca, departamento de Lima, Perú. Report to Facultad de Ciencias Natural Matemática. Lima, Perú: Universidad Nacional Federico Villarreal. Av. Río Chepén s/n El Agustino, p. 17.

[28] Sakai K, Türkay M. 2013 Revision of the genus *Ocypode* with the description of a new genus *Hoplocypode* (Crustacea: Decapoda: Brachyura). Memoirs of the Queensland Museum-Nature, 56, 665-793.

[29] Schmidt GD. 1973 Resurrection of *Southwellina* Witenberg, 1932, with a description of *Southwellina dimorpha* sp. n., and a key to genera in Polymorphidae (Acanthocephala). Journal of Parasitology, 59, 299-305. 4714596

[30] Schmidt GD. 1975 *Andracantha*, a new genus of Acanthocephala (Polymorphidae) from fish-eating birds, with descriptions of three species. Journal of Parasitology, 61, 615-20. 1165545

[31] Schmidt GD, MacLean SA. 1978 *Polymorphus (Profilicollis) major* Lundström 1942 juveniles in rock crabs, Cancer irroratus, from Maine. Journal of Parasitology, 64, 953-954.

[32] Standing MD, Heckmann RA. 2014. Features of Acanthocephalan hooks using dual beam preparation and XEDS phase maps. Poster. Submission Number 0383-00501. 2014. Microscopy and Microanalysis Meeting. Hartford. CT.

[33] Tantaleán M, Sánchez L, Gómez L, Huiza A. 2005 Acanthocephalan from Peru. Revista Peruana de Biologia, 12, 83-92.

[34] Vasquez G, Herrera Y, Martinez R, Tantaleán M. 2012. New record of Andracantha sp. (*Acanthocephala*: Polymorphidae) in the coast of Callao, Peru. Libro de resúmenes del VIII Congresso de Parasitologia.

